# Perceived stress, anxiety, and depression among university employees in Southwestern Nigeria: A cross-sectional study

**DOI:** 10.1371/journal.pmen.0000235

**Published:** 2025-01-29

**Authors:** Thomas Olumide Adeleke, Iyabo Victoria Olatubi, Grace Oluwaranti Ademuyiwa, Timothy Kayode Samson, Titilope Abisola Awotunde, Victoria Oludamola Adeleke, Adeoba Mobolaji Awolola, Matthew Idowu Olatubi

**Affiliations:** 1 Bowen University Hospital and Medical Services, Bowen University, Iwo, Osun State, Nigeria; 2 Pure and Applied Biology Programme, College of Agriculture Engineering and Science (COAES), Bowen University, Iwo, Osun state, Nigeria; 3 Public/Community Health Nursing Programme, College of Health Sciences, Bowen University, Iwo, Osun State, Nigeria; 4 Statistics Programme, College of Agriculture Engineering and Science (COAES), Bowen University, Iwo, Osun State, Nigeria; 5 Faculty of Nursing Sciences, Maternal and Child Health Nursing Programme, College of Health Sciences, Bowen University, Iwo, Nigeria; 6 Bowen University Hospital and Medical Sciences, Bowen University, Iwo, Osun State, Nigeria; 7 Anatomy Programme, Bowen University, Iwo, Osun State, Nigeria; Universiti Brunei Darussalam Pengiran Anak Puteri Rashidah Sa'adatul Bolkiah Institute of Health Sciences, BRUNEI DARUSSALAM

## Abstract

Being a centre of academic pursuits and intellectual rigour, universities frequently place a high demand on the psychological and emotional well-being of their workers. This study aims to explore the relationship between perceived stress, anxiety, and depression among employees in a university in Nigeria and explore how these stress levels are associated with anxiety and depression. We conducted a cross-sectional study in a foremost private university in Southwestern Nigeria between 28th January 2024 and 11th April 2024. The participants completed a set of self-report questionnaires measuring perceived stress, anxiety and depression symptoms, and demographic information via an electronic survey platform (Google Forms). Data was analyzed using descriptive and inferential statistics. The results showed that both perceived stress (r = 0.517, p = 0.01) and family history of heart attack (p = 0.026) were found to be significantly associated with depression (p = 0.05). The logistic regression analysis revealed that, even after adjusting for hypertension (OR = 10.43, 95% CI = 1.761–61.799), high perceived stress remained significantly associated with both anxiety (OR, 95% confidence interval (CI) = 1.761–61.799; p = .010) and depression (OR, 42.91; 95%CI = 7.557–243.605) compared with those who experienced either moderate or low levels of stress. The study showed that perceived stress is associated with anxiety and depression. Findings are expected to inform policymakers and university administrators, guiding the implementation of effective mental health support systems and stress management interventions within Nigerian universities.

## Introduction

The university environment, a hub of intellectual rigor and academic pursuit, often demands significant psychological and emotional resilience from its workforce [1,2]. Kita *et al*. [3] reported that the stress of transitioning between online and onsite teaching negatively impacted the mental health of students examined in their study. This pressure cut across every sphere of the university community; among staff, the demands of academic and administrative responsibilities; and the need to balance personal and professional lives can lead to elevated stress levels with diverse consequences.

Stress, a multifaceted response to perceived threats or demands, can profoundly impact mental health [4]. Chronic stress has been linked to a range of mental health disorders, including anxiety, depression, and burnout [5–8]. For university employees, stressors may include heavy workloads, job insecurity, overtime work, lack of resources, and the pressure to publish and secure research funding. These stressors are often compounded by external factors such as economic hardship and social instability, which are prevalent in many parts of Nigeria. Consequently, the university workforce in Nigeria may be particularly vulnerable to stress-related mental health issues.

In Nigeria, where universities are grappling with unique challenges such as underfunding [9], political instability [10], and socio-economic pressures [11], the mental well-being of the academic workforce is of paramount concern. Previous studies documented high levels of stress among university workers, especially those in the teaching categories [12] and the general university workers population [13].

Understanding the perceived stress levels among this group and their association with mental health disorders is crucial for developing targeted interventions that can enhance their well-being and productivity. Recent studies have highlighted the increasing prevalence of mental health disorders among academic staff globally [14–18], yet there is a paucity of research focusing specifically on Nigerian university workers.

This study aims to fill this gap by examining the perceived stress levels among university employees in Nigeria and exploring how these stress levels are associated with mental health disorders. By employing validated stress and mental health assessment tools, this research seeks to provide a comprehensive understanding of the factors contributing to stress and mental health challenges in this context. The findings are expected to inform policymakers and university administrators, guiding the implementation of effective mental health support systems and stress management interventions within Nigerian universities.

## Materials and method

### Study design

We performed a cross-sectional study among the staff of Bowen University, Iwo, Osun State.

### Study settings

The study was conducted among Bowen University’s workforce. Bowen University is a foremost private University owned by the Nigerian Baptist Convention. It is located in Iwo, Osun State of Nigeria. Data was collected between 28 January 2024 and 11 April 2024.

### Participants

The participants were employees from a leading privately-owned Christian university in Southwestern Nigeria. Only full-time employees who have spent at least one academic session (1 year) in the university were eligible to participate. Employees who are critically ill, on maternity leave, or sabbatical leave were excluded from the study. An electronic survey platform (Google Forms) was used to recruit participants and administer the questionnaires. The link was shared on all the university staff’s electronic platforms, mainly WhatsApp. Recruitment was conducted through WhatsApp invitations, which included a brief description of the study and a link to an informed consent document.

All participants completed a set of self-report questionnaires measuring perceived stress levels, anxiety, depression symptoms, and demographic information. Consent was obtained electronically by having participants select “Yes-Continue with Survey” to proceed or “No-Exit Survey” to terminate participation. The invitations were distributed to WhatsApp groups for junior and senior academic staff as well as junior and senior non-academic staff, encompassing a total of 1034 employees.

### Variables

The variables examined in the study were perceived stress, anxiety, and depression. The study examined the influence of perceived stress on the level of anxiety and depressive symptoms among the workers. Perceived stress, History of hypertension, diabetes, and family history of heart attack were used to predict anxiety and depressive symptoms among the participants. Perceived stress was categorized as “low”, “moderate” and “high” while anxiety and depression were categorized as “normal”, “borderline abnormal” and “abnormal”.

### Measurement

The perceived stress was measured using the Perceived Stress Scale (PSS). The PSS is a 10-item scale used to assess perceptions of general life stress. Developed in 1983, this instrument evaluates how different situations affect feelings and perceived stress. Participants rated their experiences over the last month on a 5-point scale ranging from “Never” (0), “Almost never” (1), “Sometimes” (2), “Fairly often” (3) to “Very often” (4). Scores are categorized as “Low Perceived Stress” (0–13); “Moderate Perceived Stress” (14–26) and “High Perceived Stress” (27–40).

Anxiety and Depression were measured in the study using the Hospital Anxiety and Depression Scale (HADS). The HADS, developed by Zigmond and Snaith in 1983, is a 14-item scale used to assess anxiety and depression symptoms. Participants reported their feelings over the past week. Scores are categorized as “Normal” (0–7); “Borderline abnormal” (8–10); and “Abnormal” (11–21)

### Sample size

An estimated sample of 288 workers was arrived at using the Taro Yamane formula for calculating sample size from 1034 staff on the payroll of the university. Comprising both teaching and supporting staff. A total of 275 staff members eventually participated in the study, resulting in a response rate of 95.5%.

### Ethical considerations

The study procedures were duly reviewed and approved by the Bowen University Institutional Review Board.

### Data analyses

All categorical variables were analyzed descriptively using frequency and percentage while for all quantitative variables, mean and standard deviation. The correlation between perceived stress with anxiety and depression was examined using Pearson correlation while the univariate association between categorical variables was investigated using the Chi-Square test. Also, multiple logistic regression was used to examine the association between perceived stress with anxiety and depression with and without adjusting for hypertension and a family history of heart attack. Odds ratios and their corresponding 95% confidence intervals were estimated and all analyses were carried out using the Statistical Package for Social Sciences (SPSS version 22.0).

## Results

Results showed that 46.2% of the participants were male while 53.8% were female (Table 1). The distribution of the demographics indicates that the majority of the respondents were non-teaching staff (59.3%) with most of the respondents being permanent staff (82.5%). The majority of the participants were less than 60 years old (95.9%), and Yoruba (95.7%). The mean scores of 15.47 were obtained for perceived stress with a standard deviation of 5.66 while for anxiety, and depression, the mean scores of 3.72 and 4.32 were obtained with standard deviations of 1.96 and 3.52 respectively. Based on these mean scores, it can be deduced that on average, the participants experienced moderate levels of stress, normal anxiety level, and normal depression level.

**Table 1:** Demographics of the participants (n = 275).

Variables	Number of respondents	Percentage (%)
**Sex**		
Male	127	46.2
Female	148	53.8
**Staff grade**		
Teaching	112	40.7
Non- teaching	163	59.3
**Nature of appointment**		
Contract	13	4.7
Permanent	227	82.5
Temporary	35	12.7
**Age (Years)**		
Less than 60	260	95.9
60–69	15	4.1
70 and above	0	0.0
**Ethnicity**		
Yoruba	246	95.7
Igbo	9	3.5
Hausa	2	0.8
**Highest education qualification**		
Primary School Leaving Certificate	1	0.4
Primary School Leaving Certificate	1	0.4
SSCE	2	0.7
Diploma	2	0.7
HND/ B.SC or equivalent	7	2.5
PGD	1	0.4
M.Sc	80	29.1
PhD	58	21.1
Others	124	45.1

There is a significant positive relationship between perceived stress and anxiety (r = .517, p < .01) which implies that a significant increase in perceived results in a corresponding increase in anxiety (Table 2). The result also reveals that significant positive relationship between perceived stress and depression (r = .522, p < .01) meaning that as perceived stress increases, there is an increase in depression. This therefore means that perceived stress has a significant positive relationship with both anxiety and depression (p < .05). Table 3 presents the results of the perceived stress, anxiety, and depression among the participants. The result shows that the majority of the respondents had moderate stress (63.6%), normal anxiety level (95.3%), and normal level of depression (81.1%).

**Table 2:** Correlation and mean/standard deviation for all variables (n = 275).

	Mean	SD	1	2	3
Perceived stress	15.47	5.66	1		
Anxiety	3.72	1.96	0.517**	1	
Depression	4.32	3.52	0.522**	0.624**	1

**Correlation is significant at 1% (p < .*01)*.

*Correlation is significant at 5% (p < .05).

**Table 3:** Prevalence of perceived stress, anxiety, and depression among the participants (n = 275).

	Number of respondents	Percentage (%)
**Perceived stress**		
Low stress	93	33.8
Moderate stress	175	63.6
High perceived stress	7	2.5
**Anxiety**		
Normal	262	95.3
Borderline case	13	4.7
**Depression**		
Normal	223	81.1
Borderline Case	31	11.3
Abnormal	21	7.6

The univariate association between perceived stress on anxiety and depression while also adjusting for demographic variables is presented in Table 4. Result shows that perceived stress is significantly associated with anxiety (p = 0.001, p < .01) and depression (p = 0.000, p < .01). Result also established that none of the demographic variable was found to be significantly associated with anxiety (p>.05) while only tribe shows significant association with depression (p = 0.004, p < .01) with higher prevalence of high depression among other tribes (11.1%) compared with that obtained among the three major tribes in Nigeria (Yoruba, Igbo and Hausa).

**Table 4:** Chi-Square showing an association between perceived stress, socio-demographic variables on anxiety and depression.

	Anxiety			Depression			
	Normal	Borderline case	P-value	Normal	Borderline Case	Abnormal	P-value
Perceived stress							
Low stress	93(100.0)	0(0.0)		87(93.5)	5(5.4)	1(1.1)	
Moderate stress	164(93.7)	11(6.3)	0.001**	135(77.1)	25(14.3)	15(8.6)	0.000**
High perceived stress	5(71.4)	2(28.6)		1(14.3)	1(14.3)	5(71.4)	
Sex							
Male	121 (95.3)	6(4.7)	0.998	103(81.1)	14(11.0)	10(7.9%)	0.985
Female	141(95.3)	7(4.7)		120(81.1)	17(11.5)	11(7.4)	
Staff category							
Non–teaching	153(93.9)	10(6.1)	0.299	131(80.4)	17(10.4)	15(9.2)	0.458
Teaching	109(97.3)	3(2.7)		92(82.1)	14(12.5)	6(5.4)	
Academic qualifications							
First school leaving certificates	1(100.0)	0(0.0)		1(100.0)	0(0.0)	0(0.0)	
SSCE	2(100.0)	0(0.0)	0.509	2(100.0)	0(0.0)	0(0.0)	0.897
First degree	82(94.3)	5(5.7)		73(83.9)	9(10.3)	5(5.7)	
Masters	74(92.5)	6(7.5)		64(80.0)	8(10.0)	8(10.0)	
PhD	55(96.5)	2(3.5)		42(73.7)	9(15.8)	6(10.5)	
Others	48(100.0)	0(0.0)		41(85.4)	5(10.4)	2(4.2)	
Nature of the appointment							
Contract	11(84.6)	2(15.4)	0.081	10(76.9)	2(15.4)	1(7.7)	
Temporary	35(100.0)	0(0.0)		30(85.7)	3(8.6)	2(5.7)	0.945
Permanent	216(95.2)	11(4.8)		183(80.6)	26(11.5)	18(7.9)	
Age (years)							
Less than 60	247(95.0)	13(5.0)	0.447	210(80.8)	30(11.5)	20(7.7)	0.596
60–69	11(100.0)	0(0.0)		10(90.9)	1(9.1)	0(0.0)	
Ethnicity							
Yoruba	236(95.9)	10(4.1)	0.090	202(82.1)	25(10.2)	19(7.7)	
Igbo	7(77.8)	2(22.2)		4(44.4)	5(55.6)	0(0.0)	0.004**
Hausa	2(100.0)	0(0.0)		2(100.0)	0(0.0)	0(0.0)	
Others	17(94.4)	1(5.6)		15(83.3)	1(5.6)	2(11.1)	

Result of logistic regression shows that with and without adjusting for demographics of the respondents, perceived stressed was found to be significantly associated with anxiety with an increased odds of 12.277 [C.I = 1.789–84.239] after adjusting for demographics variables and an odd ratio of 9.35 (C.I = 1.628–53.636). The Odd ratio of 12.277 indicates that the odds of anxiety is more than 12 times higher among those with higher level of perceived stress compared with those with low/moderate level of stress. For depression, the adjusted odd ratio of 39.494 [C.I = 6.009–259.567] was obtained which indicates that the odds of depression were more than 39 times higher among those that experienced high perceived stress compared with those under low or moderate level of stress. This result of the logistic regression also confirmed that perceived stress is significantly associated with both anxiety and depression (p < .05) (Table 5).

**Table 5:** Logistic regression showing association between perceived stress and mental health (anxiety and depression).

	Anxiety				Depression			
	COR (95% C.I)	P-value	AOR (95% C.I)	P-value	COR (95% C.I)	P-value	AOR (95% C.I)	P-value
Perceived stress								
Low/moderate stress	1.00		1.00		1.00			
High perceived stress	9.35 [1.628–53.636]	0.012*	12.277 [1.789–84.239]	0.000**	39.375 [7.080–218.994]	0.000**	39.494[6.009–259.567]	0.000**
Sex								
Male			1.00				1.00	
Female			1.137[0.353–3.663]	0.830			1.512 [0.547–4.181]	0.4250
Staff category								
Non–teaching			0.364[0.090–1.461]	0.154			0.410 [0.124–1.351]	0.1430
Teaching			1.00					
Academic qualifications								
First degree and below			1.141[0.337–3.861]	0.832			0.550 [0.168–0.1830]	0.3230
Post graduate degrees			1.00					
Nature of the appointment								
Temporary			1.00					
Permanent			0.848[0.160–4.492]	0.846			0.999[0.226–4.4426]	0.9999
Age (years)			0.99[40.937–1.055]	0.848			0.949[0.902–0.9999]	0.9999
Ethnicity								
Yoruba			1.00				1.00	
Non- Yoruba			0.287[0.069–1.201]	0.087			0.802[0.166–3.875]	0.9999

COR- Crude Odd ratio and AOR- Adjusted Odd ratio. Adjusted for the demographic factors

** significant at 1%(p < .01)

*significant at 5%.

## Discussion

The present study aimed to investigate the relationship between perceived stress, anxiety, and depression among employees at a privately owned faith-based university in Southwestern Nigeria. The findings revealed several important insights into the psychological well-being of university staff. The study recorded a moderate mean score for perceived stress (15.47) indicating moderate stress levels among the participants. In addition, our study also revealed a significant positive relationship between perceived stress and both anxiety and depression.

The moderate level of stress indicates that, on average, participants experienced moderate stress levels which agrees with the findings of Albikawi [19]. Our findings on the level of stress experienced by the workers are similar to what was documented among teachers in Spain [20] and university professors in Israel [12]. The level of stress among the staff in this study is lower than what was reported among academic staff in a University in Ethiopia [21]. The reason for the differences between our study and earlier study may be due to differences in the setting where the study was carried out or the fact that why our study focused on perceived stress, while theirs focused on work-related stress. Also, our study focused on both teaching and non-teaching staff of the university while most of previous studies in Nigeria focused mainly on academic staff [22]. It may also be due to innate stressors of the different populations, possibly, the university environment in Ethiopia predisposes to stress development, or the employees in Nigeria can cope better with stress due to their inherent innate characteristics and verbalize stress less than those in Ethiopia.

Interestingly, the levels of anxiety and depression were generally low among the participants in our study, with mean scores of 3.72 (SD = 1.96) for anxiety and 4.32 (SD = 3.52) for depression, suggesting normal anxiety and depression levels among the majority of the participants. This is not the case with several other findings as Fawzy and Hamed [23] recorded 87.7% in Bangladesh, 67.9% in Jordan [24], and 34.6% in Japan [25]. However, Ozamiz-Etxebarria [20] documented that levels of anxiety and depression among university teachers are low when compared to the level of stress they are exposed to.

Our findings showed that as the amount of stress that the worker is exposed to increases, their anxiety level and prevalence of depressive symptoms increase [26–30]. This is a pointer that if something is not done to arbitrate the stress that the workers are exposed it may result in increased levels of anxiety and depressive symptoms among the staff. This trend shows a great danger not only for the workers but also for the students they relate with daily as anxiety and depression have been documented to affect job performances and work output [31–33]. Furthermore, our findings showed that, even after adjusting for socio-demographic variables, high perceived stress remained significantly associated with both anxiety and depression. These findings underscore the profound impact of perceived stress on mental health, independent of other potential risk factors.

## Limitation

None randomness of the electronic survey is a limitation of this study, which might have introduced response bias. Also, perceived is not an absolute measure of stress experience which may also be a form of bias. Despite the correlations discovered, these characteristics cannot be regarded as predictors due to the intrinsic limitations of the study, which further restricts the creation of linearity. Nonetheless, the findings highlight the importance of addressing perceived stress in the workplace to mitigate its adverse effects on mental health. Interventions aimed at stress reduction could potentially decrease the prevalence of anxiety and depression among university staff.

## Conclusion

In conclusion, this study highlights the significant relationship between perceived stress and mental health outcomes among university employees. The findings suggest that moderate levels of perceived stress are common among staff and high perceived stress is significantly associated with increased anxiety and depression, independent of other health factors. Additionally, a family history of heart attack appears to be a significant predictor of depression. These insights underscore the need for targeted stress management interventions and mental health support for university employees to enhance their overall well-being. Future research should explore longitudinal data to better understand the causal relationships and develop effective strategies for stress reduction in academic settings. Also, employers and employees should devote attention to developing and suggesting through rigorous research, culturally appropriate techniques to promote stress reduction; anxiety, and depression curtailment among workers in the university environment. These techniques may include yoga, meditation, sports, exercise, dance, reiki, energy healing, spiritual lessons, etc.

## Supporting information

S1 Data (XLSX)
